# Hydrophilicity and carbon chain length effects on the gas sensing properties of chemoresistive, self-assembled monolayer carbon nanotube sensors

**DOI:** 10.3762/bjnano.10.58

**Published:** 2019-02-27

**Authors:** Juan Casanova-Cháfer, Carla Bittencourt, Eduard Llobet

**Affiliations:** 1 MINOS-EMaS, University Rovira i Virgili, Avda. Països Catalans, 26, 43007 Tarragona, Spain; 2 Chimie des Interactions Plasma-Surface (ChIPS), Research Institute for Materials Science and Engineering, Université de Mons, Avenue Copernic 1, Mons, Belgium

**Keywords:** carbon length chain, gas sensing mechanism, hydrophilicity, hydrophobicity, multiwall carbon nanotubes (MWCNTs), self-assembled monolayer (SAM), thiol

## Abstract

Here we describe the development of chemoresistive sensors employing oxygen-plasma-treated, Au-decorated multiwall carbon nanotubes (MWCNTs) functionalized with self-assembled monolayers (SAMs) of thiols. For the first time, the effects of the length of the carbon chain and its hydrophilicity on the gas sensing properties of SAMs formed on carbon nanotubes are studied, and additionally, the gas sensing mechanisms are discussed. Four thiols differing in the length of the carbon chain and in the hydrophobic or hydrophilic nature of the head functional group are studied. Transmission electron microscopy, Raman spectroscopy and X-ray photoelectron spectroscopy are used to analyze the resulting gas-sensitive hybrid films. Among the different nanomaterials tested, short-chain thiols having a hydrophilic head group, self-assembled onto Au-decorated carbon nanotubes were most responsive to nitrogen dioxide and ethanol vapors, even in the presence of ambient humidity. In particular, this nanomaterial was about eight times more sensitive to nitrogen dioxide than bare Au-decorated carbon nanotubes when operated at room temperature. This response enhancement is attributed to the interaction, via strong hydrogen bonding, of the polar molecules tested to the polar surface of hydrophilic thiols. The approach discussed here could be extended further by combining hydrophilic and hydrophobic thiol SAMs in Au-MWCNT sensor arrays as a helpful strategy for tuning sensor response and selectivity. This would make the detection of polar and nonpolar gas species employing low-power gas sensors easier, even under fluctuating ambient moisture conditions.

## Introduction

Carbon nanotubes were first observed by Sumio Iijima in 1991 [1] and since then, this nanostructure has been widely used in chemoresistive gas sensors [2–5] due to the possibility to engineer its sensitivity towards chemicals present in a local environment. One of the reasons for this is their high surface-to-volume ratio and hollow structure in which almost every single carbon atom is on the surface, making them suitable for the adsorption of gas molecules [6]. This capability to detect toxic air pollutants has made them ideal candidates for integration into different types of transducers such as chemoresistors, resonant gravimetric or field effect devices, only to cite a few applications. Bare carbon nanotubes have been employed to detect gases such as nitrogen dioxide [7], ammonia [8], oxygen [9] or ethanol [10]. However, pristine carbon nanotubes (CNTs) present some limitations for gas sensing. For example, carbon nanotube gas sensors often suffer from slow recovery, especially when operated at room temperature, which eventually results in baseline and response drift. For that reason, it is usually necessary to heat up the gas sensitive nanomaterial to higher temperatures [3] or to irradiate the sensor employing ultraviolet (UV) light, in order to promote surface cleaning. Despite these efforts, sometimes CNTs present irreversible resistance changes due to the chemisorption of gas molecules. In addition, other problems such as lack of selectivity, environmental variations (e.g., changes in humidity level) affecting sensor response, or the difficulty to detect gases characterized by low adsorption energies are often encountered [11].

In order to enhance their selectivity and/or their sensitivity, CNTs have been functionalized by introducing reactive groups onto their sidewalls, such as carboxylic acid [12–13], hydroxy [14] or carbonyl [15] groups, by decorating them with metal or metal oxide nanoparticles [10,16–19], or by creating CNT–polymer [20] or CNT–chalcogenide [21] hybrids. Employing these approaches, different carbon nanotube sensors have been reported for detecting toxic pollutants emitted from vehicle exhaust [22–23], hazardous volatile organic compounds (VOCs) [24] or chemical warfare agents (CWAs) [25–26]. Usually, these modified carbon nanotubes improve the selectivity, because the chemical specificity of bonding for target molecules is enhanced. Also sensitivity is improved via a stronger interaction between functionalized carbon nanotubes and target species.

However, apart from improved sensitivity and selectivity, the effective detection of gaseous species in the environment requires gas sensors with other specific properties, such as stability, simplicity, low-cost and fast response [6]. For that reason, the last years have seen the development of approaches in which complex molecules are grafted onto the surface of carbon nanotubes via covalent or non-covalent interactions. In such an approach, carbon nanotubes act as support and charge transport transducing elements while the recognition function is performed by grafted molecules. Two examples of this have consisted of obtaining thiol-functionalized carbon nanotube buckypapers [27] or self-assembled monolayers (SAMs) of thiol molecules onto Au-decorated CNTs [28], in order to enhance the properties required to detect toxic gases. Additionally, the electronic properties of single-wall CNTs heavily depend on chirality, and the conduction properties of single-wall CNT films on top of interdigitated electrodes change dramatically depending on whether metallic nanotubes are above or below the percolation threshold [29–30]. In contrast, multiwall CNT mats always present a mild p-type semiconductor behavior which improves device to device reproducibility without the added burden of sorting nanotubes according to their metallic or semiconducting character before being integrated in gas sensing devices.

In view of developing sensitive, fast-responding, low power consumption and more selective sensors towards nitrogen dioxide or ethanol, in this paper, we combine oxygen plasma treated, Au-decorated MWCNTs with different thiols (see Figure S1, Supporting Information File 1). While thiol molecules behave as a chemoselective material responsible for the recognition of gas species, carbon nanotubes act as efficient charge transport networks, enabling the implementation of a chemoresistive transduction. Different thiols were attached to the gold nanoparticles creating SAMs (see Figure S2, Supporting Information File 1), which differed in their terminal functional groups and in the length of their carbon chain. This enables studying the effects of hydrophilicity or hydrophobicity of terminal functional groups and carbon chain length on the gas sensing properties of SAMs supported on CNTs. The response towards two gases, nitrogen dioxide and ethanol, was investigated. On the one hand, the detection of nitrogen dioxide has attracted great interest because of its adverse consequences for the environment and health risks associated to exposure for humans [18]. In environmental monitoring applications, nitrogen dioxide should be detected in the 20 to 200 ppb range. Some authors have reported the detection of NO_2_ at such low concentrations using sensors employing carbon nanotubes [31–32]. In addition, sensors can be employed as well to determine nitrogen oxide emission from combustion engines. In this case, the concentrations to be measured range in the tens to hundreds of ppm [33–34]. On the other hand, ethanol sensors can be used in the automotive sector to check the concentration of ethanol in fuel blends, especially in biofuels. Ethanol is added to fuels since it can act as a radical scavenger, improving air quality by diminishing the concentration of pollutants emitted from combustion engines. However, an excessive amount of ethanol in the blend can damage automotive fuel lines [35]. Additionally, by testing oxidizing (nitrogen dioxide) and reducing (ethanol) species, it is possible to obtain more information about the gas sensing behavior of functionalized carbon nanotube mats. Moreover, the effect of ambient moisture on the chemical response was studied, determining how moisture interference depended on the characteristics of the different thiols considered. Finally, the mechanisms for the interaction between gas molecules and the hybrid nanomaterials, which explain the experimental results obtained with the different sensors tested, are introduced and discussed.

## Results and Discussion

### Material characterization

**Figure 1:**
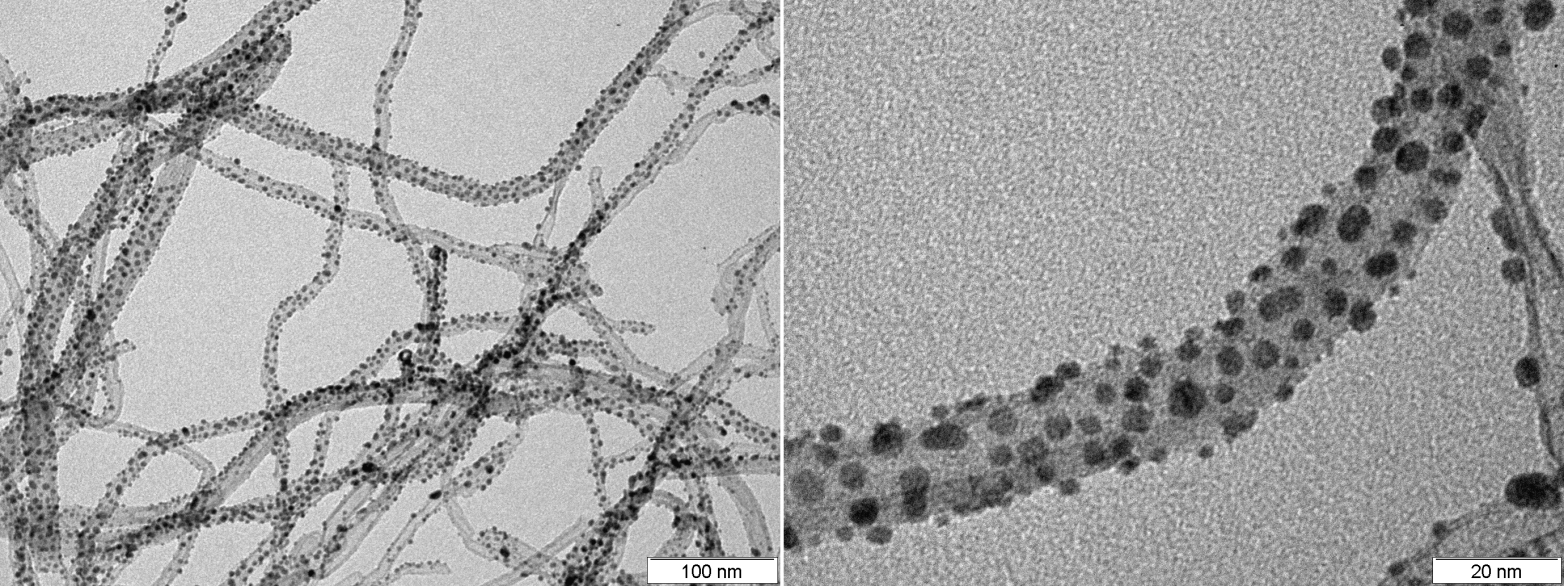
TEM images of MWCNTs decorated with gold nanoparticles.

MWCNTs decorated with Au nanoparticles were analyzed by TEM in order to observe the distribution of metal nanoparticles on the carbon nanotubes. Figure 1 shows that the CNT sidewalls are densely and quite homogeneously decorated with Au nanoparticles (Table S1 in Supporting Information File 1 shows quantitative XPS results indicating that the Au content was 5.7 wt % in these samples). A monomodal distribution of Au nanoparticles is obtained with average diameter of about 4 nm. The gold nanoparticles appear very close one to another (typically 10 nm apart), which will affect the gas sensing mechanism, as will be discussed in detail below.

The crystallinity of oxygen-plasma-treated MWCNTs decorated with gold nanoparticles was characterized by Raman spectroscopy. Taking the intensity ratio of the D/G bands into consideration, the material presents a low level of crystallinity with defects caused by the oxygen plasma treatment. However, the presence of such defects in CNTs is essential, since it has been shown that they play the role of nucleation centers and help to anchor the Au nanoparticles during the sputtering process [36]. In other words, the defects help to achieve a dense and homogenous decoration of CNT sidewalls with Au nanoparticles, preventing their mobility and coalescence.

**Figure 2:**
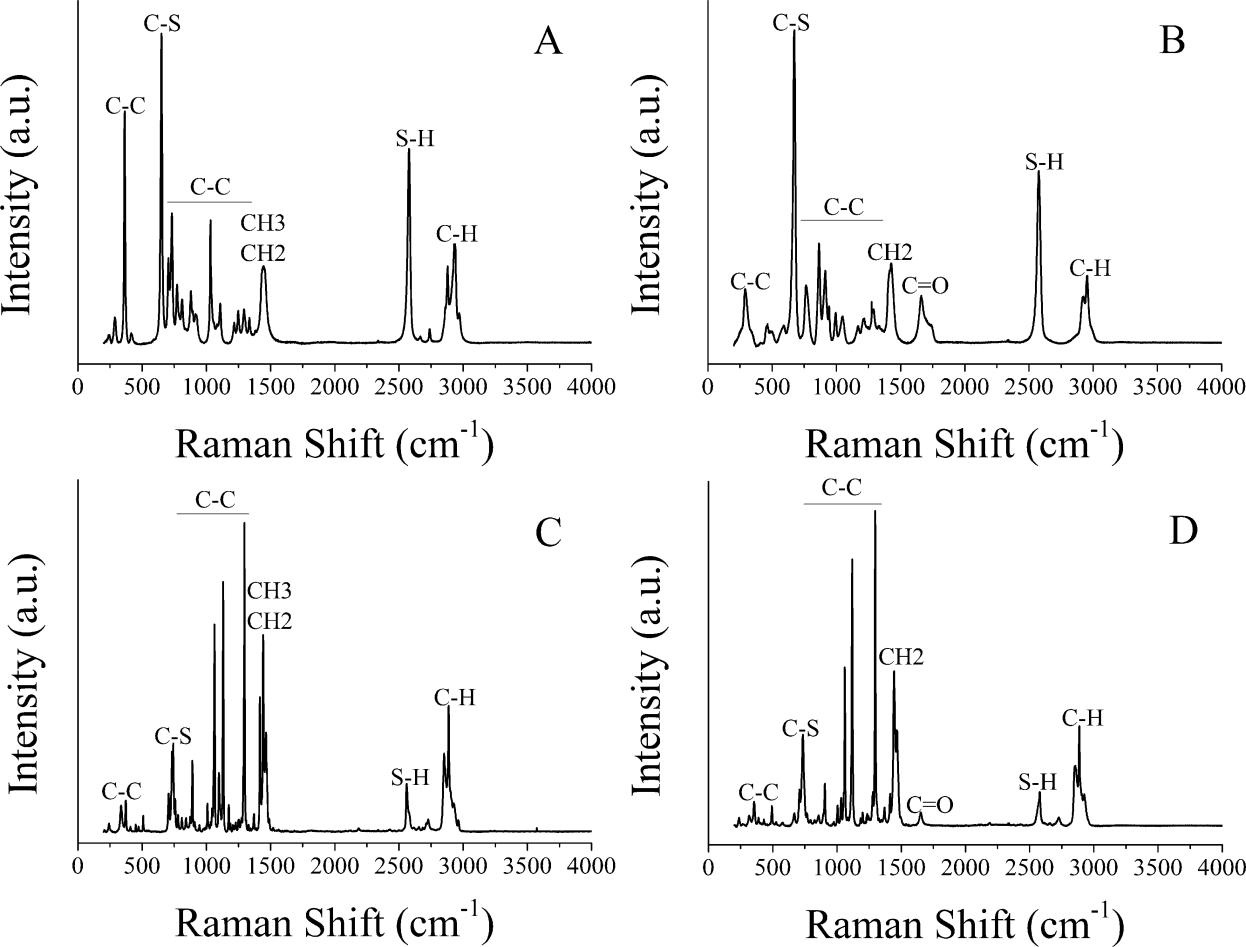
Raman spectra for the different thiols studied. A) C_3_H_8_S; B) C_3_H_6_O_2_S; C) C_16_H_34_S; D) C_16_H_32_O_2_S.

The different thiols employed in this work were characterized by Raman spectroscopy (see Figure 2). A Peltier cell was used in order to keep samples at 4 °C and stabilize them, because unbound thiols present high volatility, even at room temperature. The Raman analysis of the thiols shows the presence of characteristic peaks in all samples related to the mode of vibration of aliphatic carbon chains at wavenumbers between 250–400 cm^−1^ and 630–790 cm^−1^. Other important bands can be found at the following wavenumbers: 735 cm^−1^, 2580 cm^−1^ and 2900 cm^−1^, which correspond to C–S, S–H and C–H elongations, respectively. In addition, hydrophilic thiols (i.e., C_3_H_6_O_2_S and C_16_H_32_O_2_S) present a characteristic peak at 1680 cm^−1^ attributed to C=O elongation. In contrast, hydrophobic thiols (C_3_H_8_S and C_16_H_34_S) show a characteristic peak at 1440 cm^−1^ related to CH_2_ and CH_3_ radicals. Note that O–H elongations do not appear in the Raman spectra, probably due to the weak intensity of the O–H peak in Raman spectroscopy.

**Figure 3:**
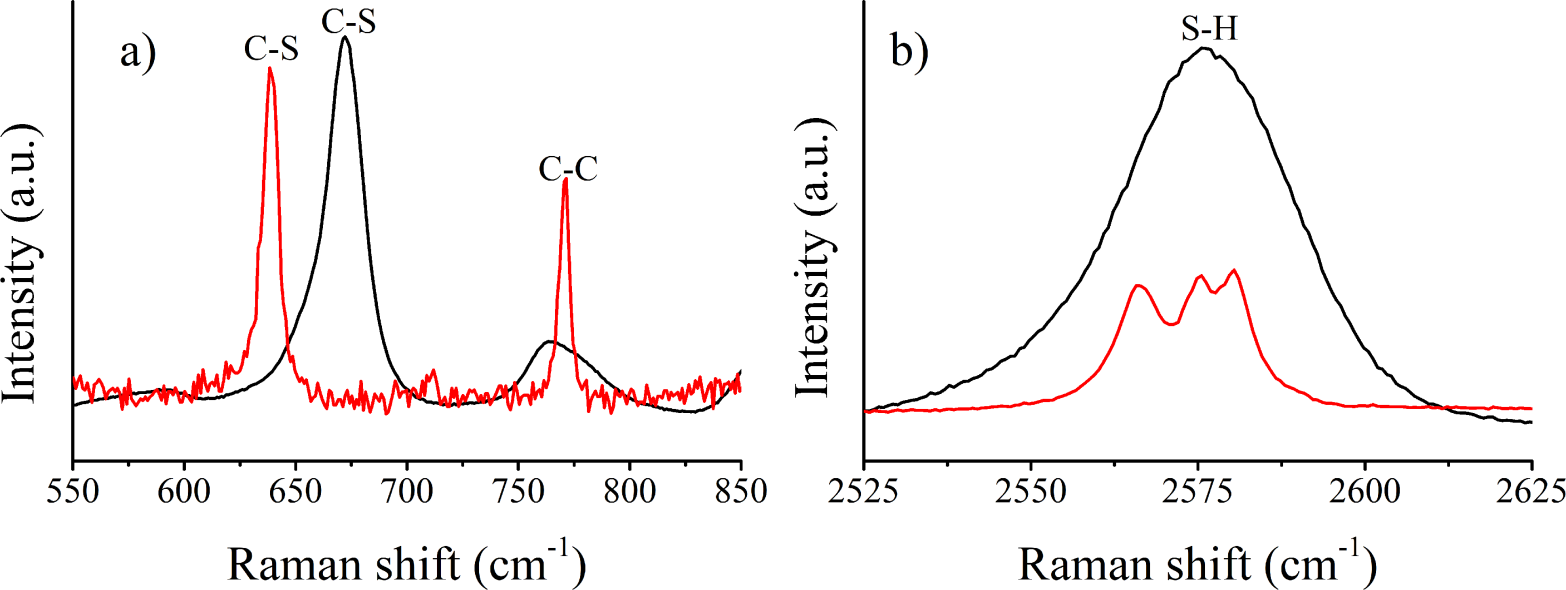
a) Comparison between unbound (i.e., free) thiol and hybrid SAM-MWCNTs for C–S and C–C elongations, b) comparison between unbound thiol and hybrid SAM-MWCNTs for S–H elongations. Black line: thiol (C_2_H_6_O_2_S); red line: thiol-Au-MWCNTs.

Once these thiols had been attached to Au-MWCNTs via the SAM technique, it was not possible to detect many of their most characteristic peaks in the resulting hybrid nanomaterials, because the high intensity peaks from MWCNTs had a masking effect in the survey spectra. For this reason, specific regions of the Raman spectrum in which no peaks from MWCNTs appear were acquired in order to confirm the presence of the thiols. Figure 3a shows the region between 550 and 850 cm^−1^ providing information about the C–S and C–C elongations. The first peak appears at 638 and 670 cm^−1^ for Au-MWCNT bound and free C_3_H_6_O_2_S, respectively. Two main aspects may be responsible for this shift. First, the temperature during the acquisition of the spectra was different. While free C_3_H_6_O_2_S was kept at 4 °C to avoid the fast volatilization that would have happened at room temperature, cooling was not necessary when thiols were attached to Au-MWCNTs and, in this case, the acquisition was conducted at room temperature. Second, the environment of these molecules was absolutely different. In other words, despite the fact that what is detected in both cases is the C–S elongation, the sulfur in free C_3_H_6_O_2_S was bonded in addition to a hydrogen atom, while in thiol attached to Au-MWCNT samples, the sulfur was bonded to a gold atom. Moreover, a second peak can be observed at 770 cm^−1^ indicating the presence of aliphatic carbon chains. This peak is a proof of presence of thiols on the surface of gold decorated CNTs because this C–C elongation is also registered for unattached thiols. In addition, this peak cannot be attributed to MWCNTs because the vibrations of the aromatic ring carbon chain appear in the region near 1500 cm^−1^.

Figure 3a confirms the presence of thiols in thiol-Au-MWCNT samples, however, Figure 3b shows that some of these thiols remain unattached to Au. The 2550–2600 cm^−1^ region was analyzed in order to find the strong peak corresponding to S–H elongations, which is indicative of the presence of unbound thiols. The intensity of this peak in thiol-Au-MWCNT samples is significantly lower than that found in free thiol samples (both spectra in Figure 3b were measured under identical experimental conditions). The fact that a small peak remains in thiol-Au-MWCNT samples is indicative that basically a SAM is formed, but some thiol molecules remain unattached to gold nanoparticles, despite the cleaning step implemented after the formation of the SAM.

In Figure 3, a clear localized surface plasma resonance (LSPR) effect (e.g., enhancement in signal intensity) cannot be observed. The reason could be due to the extremely low power of the laser used to record the Raman spectra, in order to prevent damaging the SAMs. As a consequence, a possible explanation is that the applied energy was not enough to create an important electromagnetic field around the Au–nanoparticle–dielectric interface. The Raman shift observed can be caused, in part, by LSPR. Further details can be found in Supporting Information File 1 (Figure S3).

**Figure 4:**
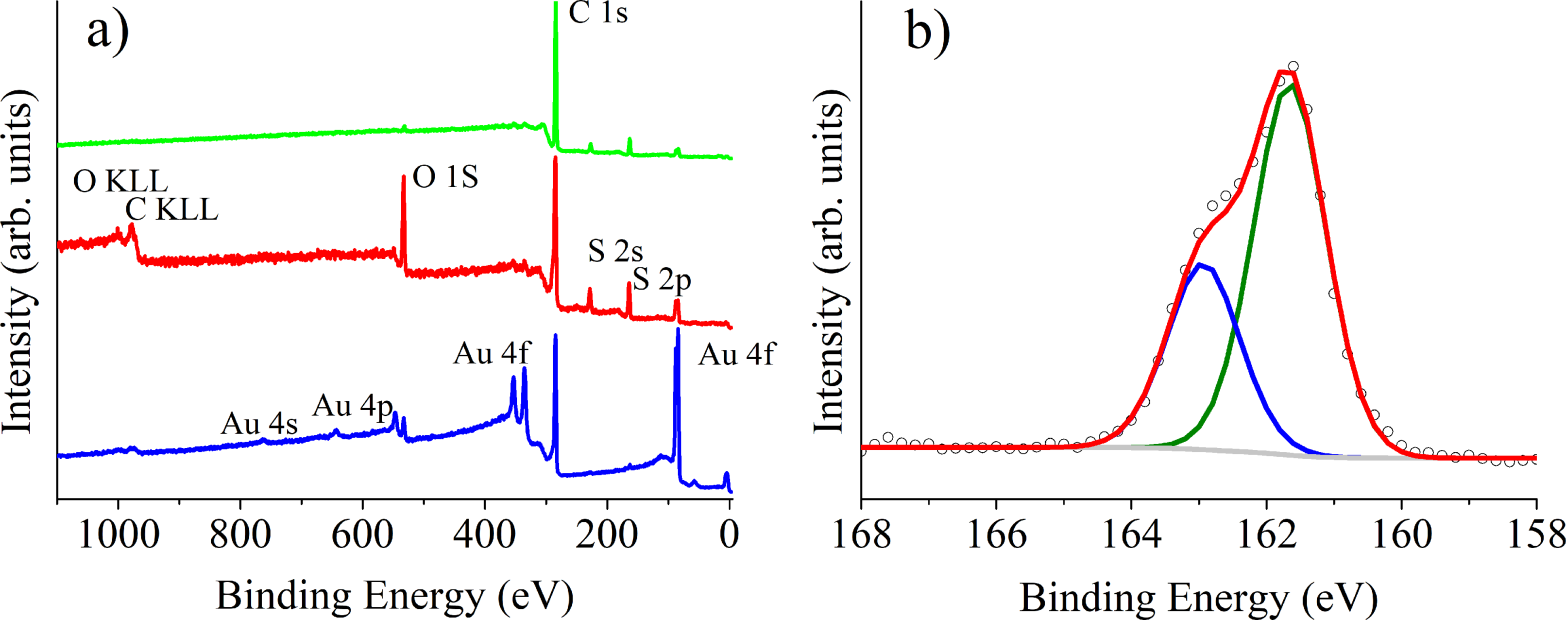
(a) X-ray photoelectron survey spectra recorded on Au-CNTs (blue), short-chain thiol-Au-CNTs (red) and long-chain thiol-Au-CNTs (green) samples. (b) S 2p spectrum acquired on a thiol-Au-MWCNT sample. The results for a short-chain thiol (C_3_H_8_S) are shown here, as a typical example of the SAMs obtained.

Since oxygen-plasma-treated CNTs were employed, a study on the nature of oxygenated species present (acting as nucleation centers for the anchoring the Au NPs) was conducted using XPS. The chemical modification caused by the plasma treatment results in the presence of hydroxy, carbonyl and carboxyl groups [36]. Furthermore, Au nucleation centers occur mainly in the proximity of oxygenated defects created during the plasma treatment [37]. These results are summarized in Supporting Information File 1, Figure S4 and Table S1. The presence of thiols attached to Au NPs was further confirmed by XPS analysis. Figure 4a shows the comparison of the XPS survey spectra recorded on the samples and a reference (gold on CNTs). In the reference sample, the presence of only gold and carbon related peaks is clear; after the reaction with thiols, we can observe the presence of peaks generated by photoelectrons emitted from sulfur atoms for both samples and oxygen atoms for the short-chain thiol. In order to investigate the nature of the bonding of the thiols we investigated the chemical shift of the S 2p core level. The S 2p spectra acquired on the samples show a doublet structure that can be ascribed to the S 2p_3/2_ and S 2p_1/2_ peaks. All spectra were fitted using a 2:1 peak area ratio and a 1.2 eV splitting, as shown for a short-chain thiol sample in Figure 4b. The S 2p_3/2_ peak centered at 161.9 eV is reported to be generated by photoelectrons emitted from sulfur atoms bound to gold atoms at the gold nanoparticle surface as thiolate species [38]. The nonexistence of peaks in the binding energy region above 164 eV suggests the absence (or a non-detectable amount) of unbound thiol molecules (S 2p_1/2_ BE ≈ 165 eV), which confirms the correct formation of SAMs. The small amount of unbound thiol molecules detected by Raman spectroscopy were possibly removed under the ultrahigh vacuum needed to perform XPS, which would explain that only thiols attached to Au were detected employing this technique. Table S1 in Supporting Information File 1 reports quantitative XPS results performed before and after the formation of the SAM. These results indicate the almost complete coverage of Au nanoparticles with thiols, since Au (which accounted for 5.7 wt % in bare samples) is almost no longer “visible” by XPS after the formation of the SAM.

The hydrophilicity of the different coatings was studied via contact angle measurements. It was found that bare Au-MWCNTs showed a hydrophilic character due to the presence of carboxyl groups grafted to their sidewalls. Samples functionalized with SAMS having –COOH head groups showed an increased hydrophilic character and samples functionalized with SAMs having –CH_3_ head groups possessed a clear hydrophobic character. These results are summarized in Supporting Information File 1 (see Figure S5).

### Gas sensing results

Four different thiols were immobilized, via the SAM technique, on Au-decorated MWCNT mats to obtain gas sensors. The differences between the thiols chosen were in the length of their carbon chain and in their terminal functional group. Two thiols with short-length carbon chains (3 carbons) and two with long-length carbon chains (16 carbons) were used. Moreover, for each one of these two lengths, either a hydrophilic functional group (–COOH) or a more hydrophobic one (–CH_3_) was selected. Therefore, the following thiols were finally employed to form self-assembled monolayers: 3-mercaptopropanoic acid (C_3_H_6_O_2_S) and 16-mercaptohexadecanoic acid (C_16_H_32_O_2_S) as hydrophilic molecules, and 1-propanethiol (C_3_H_8_S) and 1-hexadecanethiol (C_16_H_34_S) as more hydrophobic molecules. Additionally, control sensors were produced, which integrated bare Au-decorated MWCNTs, in order to better assess the effect of the thiol SAMs on the gas sensing properties of nanomaterials.

Gas measurements were performed under dry air. It is well known that the triple bond in molecular nitrogen makes it chemically inert, however, oxygen presents high electronegativity (3.44 in Pauling scale) making it quite reactive with the sensor surface. Oxygen acts as an electron acceptor due to its lone pairs of valence electrons and can be adsorbed on the sensor surface, p-doping CNTs [39]. To correctly identify the response towards target species (i.e., nitrogen dioxide and ethanol), the oxygen concentration was kept constant at 21% throughout the measurement process. The adsorption of oxygen may result in a slight oxidation of the thiols on the long term.

The sensors were exposed to different concentrations of nitrogen dioxide (NO_2_) and ethanol (C_2_H_5_OH). The exposure time to gases or vapors was set to 5 minutes in order to obtain a clear enough response for sensors operated at room temperature, which reduces power consumption and increases the sensor lifespan. The sensors were allowed to regain their baseline resistance by flowing dry air through the sensor chamber. Measurements (exposures) were repeated at 4 h intervals. Both during their exposure to the target species (response) and to dry air (recovery), the sensors were operated at room temperature. No heating or UV irradiation was applied to speed up detection and recovery processes. The sensor response (%) is defined as ((*R*_G_ − *R*_0_)/*R*_0_) × 100.

**Figure 5:**
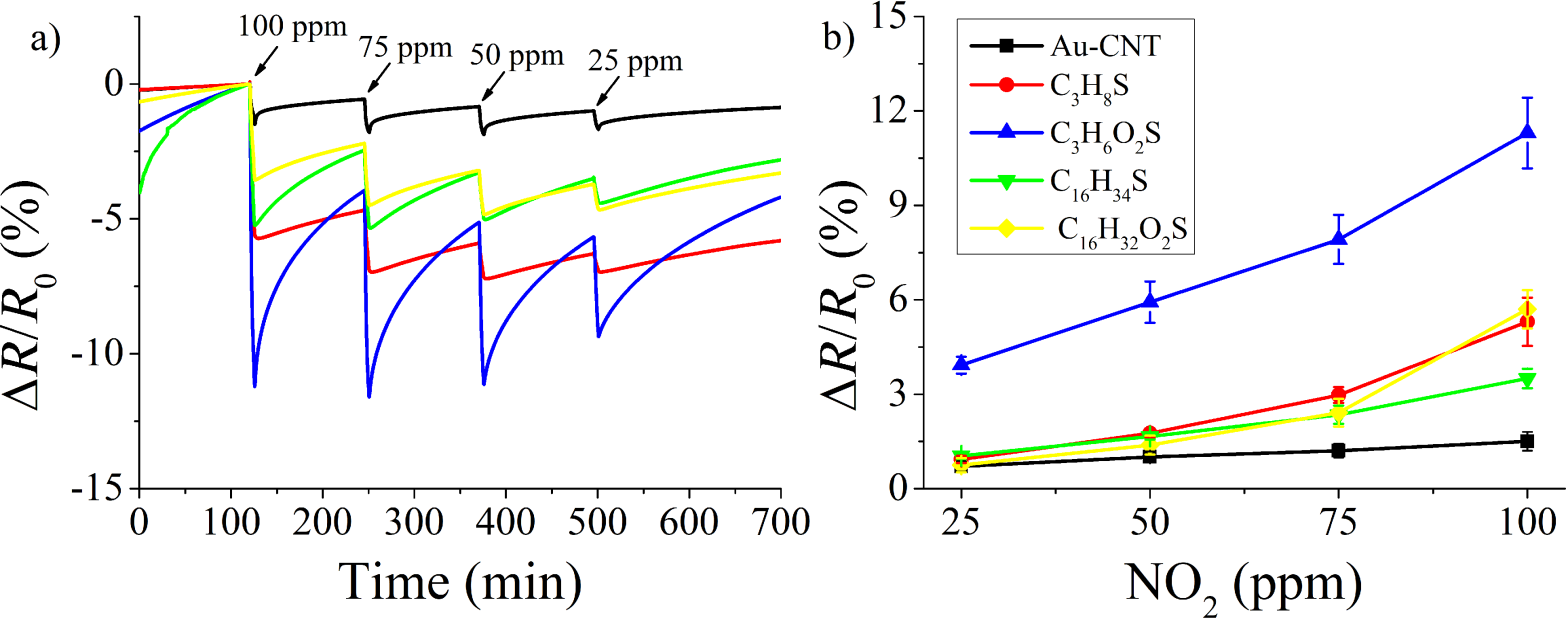
a) Sensor response to nitrogen dioxide exposure and recovery cycles in air, and b) calibration curves for the different nitrogen dioxide concentrations tested.

Nitrogen dioxide is a strong oxidizing agent that acts as an electron acceptor and presents electrophilic properties, which allow these molecules to be adsorbed onto the sensor surface. A control Au-MWCNT sensor and hybrid sensors (SAMs attached on Au-MWCNTs) were measured at four different nitrogen dioxide concentrations. The dynamic response and recovery curves are shown in Figure 5a. The control Au-MWCNT sensor showed the lowest responsiveness to nitrogen dioxide among the different sensors tested. This is in agreement with previous results in which oxygen-plasma-treated CNTs were more responsive to nitrogen dioxide than Au, Pt or Pd-decorated CNTs when operated at room temperature [10,18,40]. This was attributed to a stronger interaction and charge transfer between nitrogen dioxide and oxygenated defects in CNTs than with metal clusters. Possibly the surface of metal clusters requires higher operating temperatures to act as reactive sites for the adsorption of nitrogen dioxide molecules. Therefore, the response obtained for Au-MWCNTs cannot be explained by the catalytic activity of gold clusters at the nanometer range, and instead the resistance changes observed are probably based on the interaction between the carboxylic acid functional groups in MWCNTs and nitrogen dioxide. In contrast, the response to nitrogen dioxide is clearly enhanced (up to an 8-fold increase) when hybrid materials formed by thiol SAMs on Au-MWCNTs are used. Since it was found that Au nanoparticles were completely covered by the SAM of thiols, we assume that thiol–gas interactions dominate the response observed. In other words, the Au nanoparticles for thiol SAMs in Au-MWCNT sensors do not play a significant role in gas detection.

**Figure 6:**
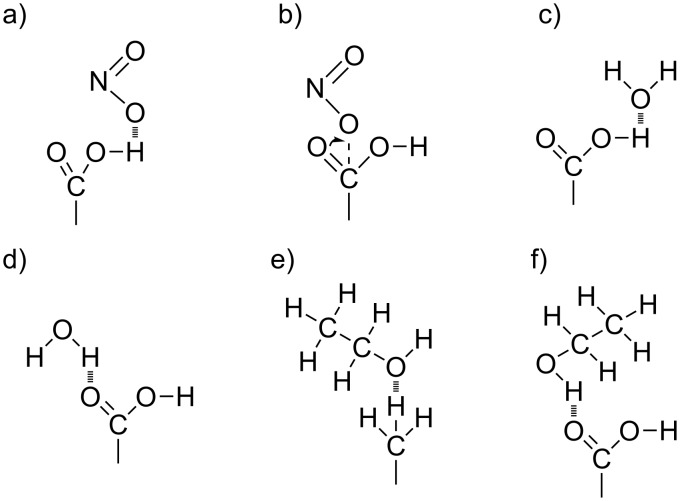
Electrostatic interactions pathways between –COOH functional groups and nitrogen dioxide (a, b), water (c, d) and ethanol (e, f).

The results show that short length thiols having a hydrophilic head group present higher response and sensitivity to nitrogen dioxide than any other SAM tested (see Figure 5b). A hypothesis to explain this result is based on two main effects. The first one is due to the hydrophilic properties of the surface, given the presence of carboxylic acid in the head group of hydrophilic SAMs. This favors the occurrence of an electrostatic interaction between the nitrogen dioxide molecule and the SAM following two possible pathways. Namely, hydrogen bonding between COOH and nitrogen dioxide molecules (see Figure 6a) and covalent interaction (see Figure 6b). The second effect is related to the length of the carbon chain and, in consequence, to the concentration of carboxyl groups that are accessible to nitrogen dioxide molecules. When short length carbon chain thiols are used (see Figure S2a, Supporting Information File 1) a vertically aligned SAM can be easily formed. Under this configuration, high sensitivity to nitrogen dioxide is obtained because the molecule can interact not only with the COOH groups in the heads of the SAM, but also nitrogen dioxide can diffuse into the SAM and eventually react with the carboxyl groups present on the surface of MWCNTs. In contrast, for SAMs employing long carbon chains (i.e., 16 carbon atoms), such molecules probably collapse and overlap on the surface of CNTs (see Figure S2b, Supporting Information File 1), making it more difficult for gas molecules to access the COOH groups on carbon nanotubes via a steric hindrance effect. This would explain why hydrophilic SAMs with long carbon chains present lower response towards nitrogen dioxide in comparison to hydrophilic, short carbon chain SAMs. In addition, overlapped SAMs can result in the formation of carboxylic acid dimers [41], further decreasing sensitivity.

On the other hand, hydrophobic SAMs present limited responsiveness towards nitrogen dioxide. The fact that the resistive response is far lower than that of hydrophilic SAMs is indicative of the weak chemical affinity between the polar nitrogen dioxide molecule and the methyl functional groups of hydrophobic SAMs. The nature of these interactions is still the subject of debate. While weak intermolecular attractive forces could be responsible for these interactions, some authors maintain that the formation of hydrogen bonds between the methyl groups present in the thiol molecules and the gas species cannot be ruled out [42]. While hydrophilic functional groups (e.g., COOH) present high surface energy due to their high affinity towards polar molecules, such as nitrogen dioxide, hydrophobic functional groups (e.g., CH_3_) present low surface energy due to their low affinity to polar molecules [43]. The low affinity of hydrophobic thiols towards polar gases explains the lower response observed. Similarly, short-chain thiols present a slightly higher response to nitrogen dioxide than long-chain thiols. This can be attributed, once more, to the molecule being able to diffuse into the SAM of short-chain thiols and reach carboxyl groups present on the surface of CNTs.

With respect to sensor recovery in dry air after a nitrogen dioxide detection event, it was observed that it was more difficult for hydrophilic thiols, especially short carbon chain thiols to regain their baseline resistance than for hydrophobic ones. Probably the cause of this effect is due to the stronger electrostatic interactions between nitrogen dioxide and hydrophilic thiols, leading to the formation of stronger hydrogen bonds.

**Figure 7:**
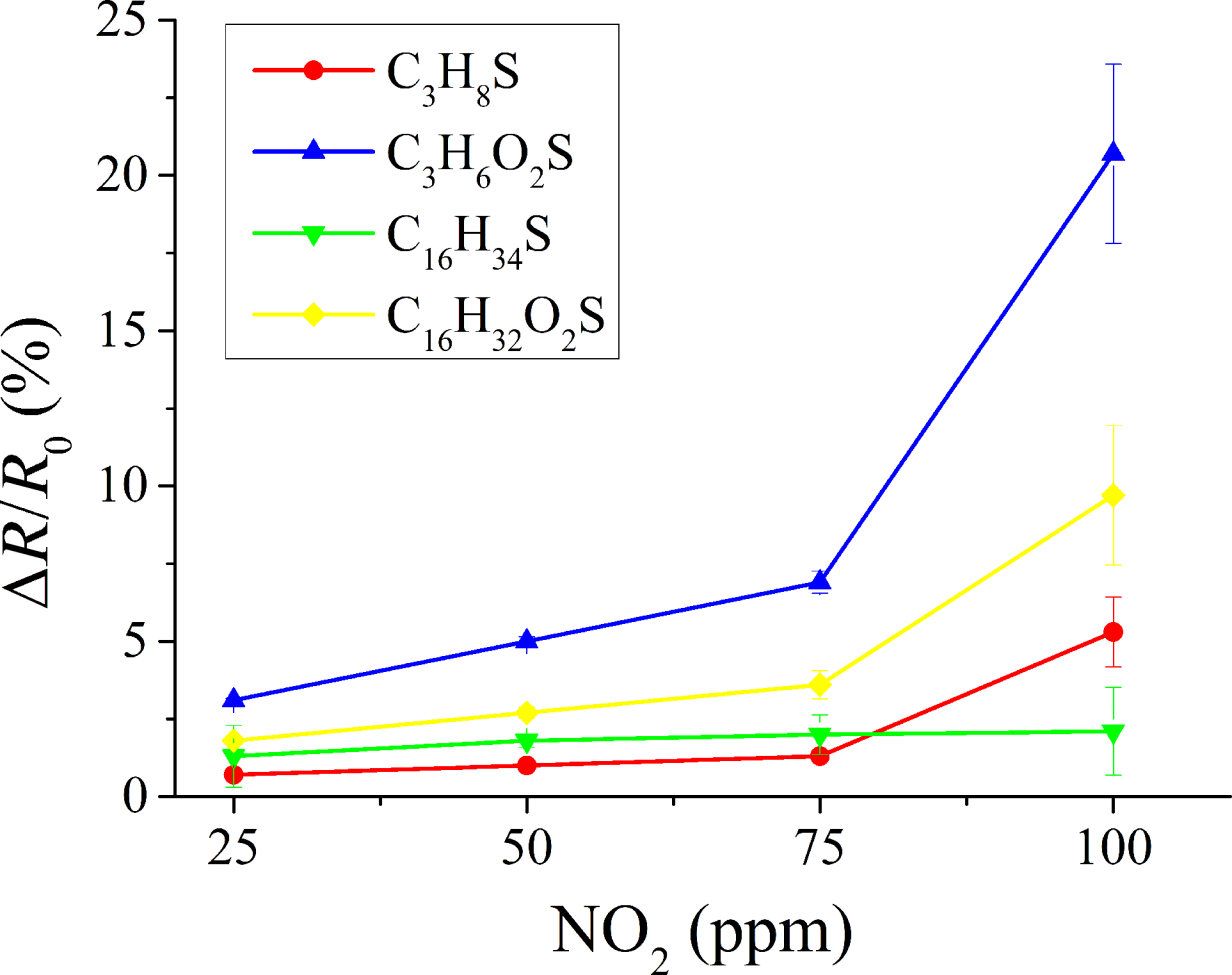
Calibration curves for different concentrations of nitrogen dioxide under humid conditions (50% R.H.).

The effect of ambient moisture on the sensor response was studied. In order to do so, the gas flow was humidified to 50% relative humidity (at 20 °C) and the same nitrogen dioxide concentrations that had been studied previously under dry conditions were measured again. These results are shown in Figure 7. As it could be expected, the presence of ambient moisture increases the sensitivity to a polar gas like nitrogen dioxide for sensors employing hydrophilic thiols [44]. In contrast, the response to nitrogen dioxide remains basically unchanged for sensors employing hydrophobic thiols. Ambient moisture can interact with the sensor surface via hydrogen bonds (see Figure 6c,d). However, the COOH groups (in hydrophilic thiols) present higher affinity to H_2_O than the CH_3_ groups (in hydrophobic thiols). The response of carbon nanotubes functionalized with short-chain, hydrophilic thiols towards NO_2_ increases in humid conditions. While at the lower concentrations tested (i.e., 25 to 75 ppm) the response is almost unaffected by changes in the background humidity; at 100 ppm, nearly a two-fold increase is observed. In hydrophilic thiols, once the saturation of free COOH groups present on the surface of the sensor is reached (because both water and nitrogen dioxide molecules get adsorbed), a water-mediated adsorption of nitrogen dioxide takes place [45], which would explain the highly nonlinear increase in response observed for the highest measured concentration of nitrogen dioxide [46]. Since the presence of ambient moisture only mildly affects the response of short-chain hydrophilic thiols towards the lower concentrations of nitrogen dioxide, in a real application these effects could be compensated for via the use of a humidity sensor and an appropriate calibration.

The presence of an adsorbed water layer on hydrophilic thiol sensors and the reported water-mediated adsorption of nitrogen dioxide could explain the improved response to humid nitrogen dioxide observed for such sensors. The absence of this adsorbed water layer on hydrophobic thiol sensors could explain the fact that, for such sensors, their response to nitrogen dioxide remains basically the same under dry or humid conditions. Similarly to the case in which nitrogen dioxide was detected under dry conditions, short-chain hydrophilic thiols present a higher response than long-chain thiols under humid conditions. This behavior can be attributed to the same reasons already discussed for the dry detection of nitrogen dioxide.

**Figure 8:**
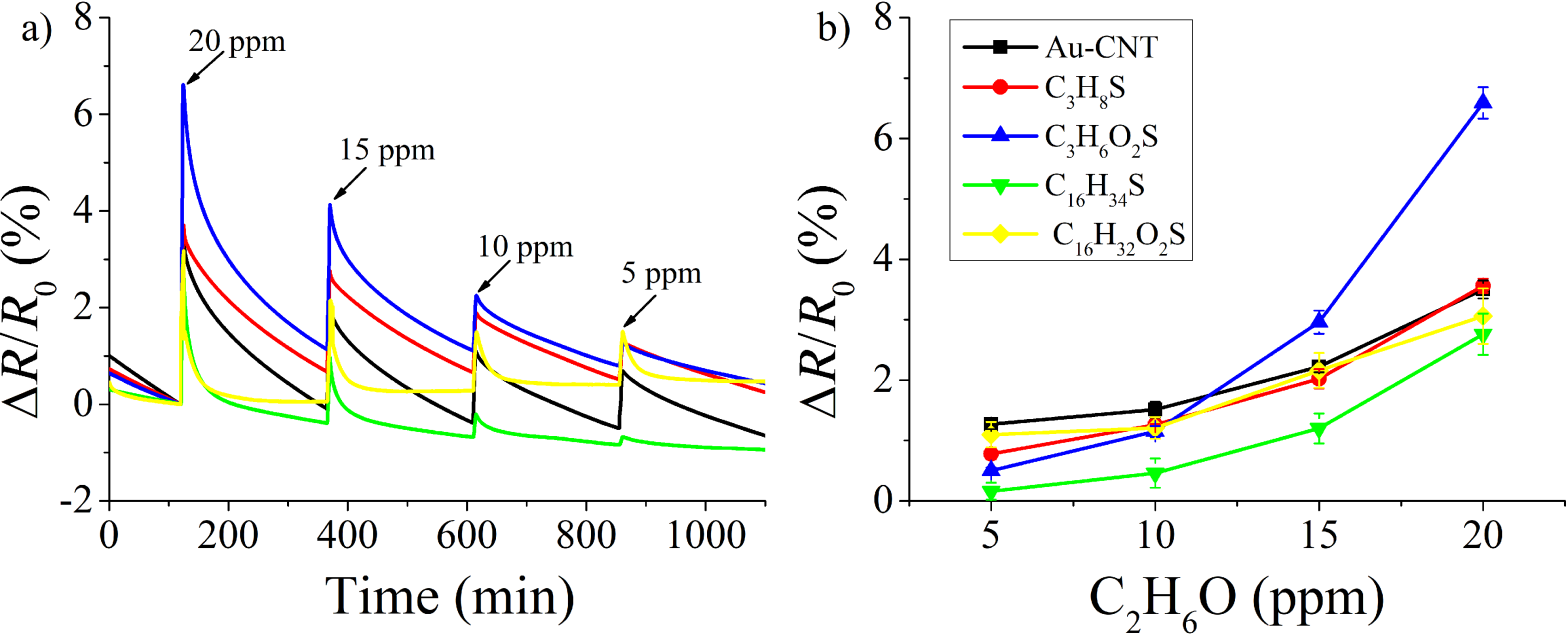
a) Sensor response to ethanol exposure and recovery cycles in air. b) Calibration curves for the different ethanol concentrations tested.

A similar experimental approach was employed to study the response towards ethanol vapors. These results are summarized in Figure 8, where it can be observed that the highest sensitivity towards ethanol (i.e., slope of the calibration curves shown in Figure 8b) is obtained for the hydrophilic SAM having a short length carbon chain. The gas sensing mechanism for detecting ethanol is, once more, related to hydrogen bonding (see Figure 6e,f). It is well-known that hydrophilic groups present more affinity to polar compounds (e.g., ethanol) than hydrophobic radicals. This concept can explain why the short hydrophilic chain thiol presents higher response to ethanol vapors, while the other SAMs show similar responses with very little differences. The absence of carboxyl groups in hydrophobic thiols and the combined effects of steric hindrance and formation of carboxylic acid dimers in long-chain, hydrophilic thiols, as already discussed for nitrogen dioxide, could explain the lower ethanol response and sensitivity observed. Finally, the reference sample (i.e., Au-MWCNTs) presents low response and sensitivity to ethanol, showing similar values to those of hydrophobic or long-chain thiols. This is indicative that the mechanism of interaction with ethanol would mainly involve carboxyl groups present on the surface of CNTs. On the other hand, gold nanoparticles probably present a residual contribution to ethanol response, due to these nanoparticles having low affinity to ethanol molecules and remaining catalytically inactive at room temperature.

The extent of energy exchange between gas molecules and the surface of the sensors depends on surface rigidity and mass and also on the forces between the impinging gas molecules and the surface functional groups. Morris and co-workers employed ab initio calculations to estimate the interaction energies between both polar and nonpolar gases with polar (OH-terminated) and nonpolar (CH_3_-terminated) SAMs [47]. They concluded that interaction forces spanned over a wide range from strong hydrogen bonding between polar gases and polar SAMs to weak dispersion forces for nonpolar gases and nonpolar SAMs. In particular, they found that the magnitude of the interaction energy between methane (nonpolar) and polar or nonpolar terminated SAMs was low and not significantly different. In contrast, the interaction energies between water (polar) and nonpolar or polar-terminated SAMs exhibited a more than one order of magnitude difference in favor of polar SAMs.

Taking into account these theoretical results, strong hydrogen bonding interactions can be assumed between the carboxyl terminals of hydrophilic thiols and the polar species studied here (i.e., ethanol vapors or nitrogen dioxide). This results in high sensor response at room temperature and difficulties for sensors to regain their baseline values. This interaction is weaker with the methyl terminals of hydrophobic thiols, which translates into lower sensor response. In that sense, the combination of hydrophilic and hydrophobic short-chain SAMs in a sensor array should be helpful for detecting polar and nonpolar species and to fight ambient moisture interference.

Finally, some examples of functionalized and pristine MWCNTs employed in gas sensors operating at room temperature are summarized in Table 1, showing a comparison of sensitivities obtained in similar approaches.

**Table 1:** Response ((Δ*R*/*R*_0_) × 100) to nitrogen dioxide and ethanol with differently functionalized MWCNTs. N/T: Not tested.

Sample	NO_2_/RT^a^	Ref.	Ethanol/RT^a^	Ref.

SAM/Au/MWCNT	11.4 (100 ppm)/5 min	this work	6.4 (20 ppm)/5 min	this work
Au/MWCNT	1.5 (100 ppm)/5 min		3.5 (20 ppm)/5 min	
SAM/Au/MWCNT	N/T	–	22 (20 ppm)/30 min	[28]
MWCNT	10.8 (100 ppm)/5 min	[21]	0.4 (100 ppm)/5 min	[48]
coated Au MWCNT	N/T	–	1.2 (50 ppm)/5 min	[48]
Au/MWCNT	12.0 (6.5 ppm)/20 min	[49]	0.4 (50 ppm)/15 min	[36]

^a^RT = reaction time, is defined as the exposure time to a target gas.

## Conclusion

This paper describes the development of chemoresistive sensors employing oxygen-plasma-treated, Au-decorated MWCNTs functionalized with self-assembled monolayers of thiols. The four different thiols studied differ in the length of their carbon chain (either 3 or 16 carbons) and in the head group (either carboxylic acid or methyl). This has enabled the study of the effects of hydrophilicity or hydrophobicity of terminal functional groups and carbon chain length on the gas sensing properties of SAMs supported on CNTs. Under this approach, thiols should, in principle, behave as a chemoselective material responsible for the recognition of gas species. In contrast, carbon nanotubes should not play a major role as receptors for target gas molecules, but instead act as efficient charge transport networks, enabling the efficient implementation of chemoresistive sensors.

It was found that short-chain thiols having a hydrophilic head group (carboxylic acid) were the most responsive to the oxidizing (nitrogen dioxide) and reducing (ethanol vapors) tested. This was attributed to the interaction, via strong hydrogen bonding, of the polar molecules tested to the polar surface of hydrophilic thiols. The contribution to the sensor response of oxygenated defects present on the surface of carbon nanotubes could not be ruled out for CNTs functionalized with short-chain thiols. However, this contribution should be moderate, since Au-MWCNTs functionalized with hydrophilic short-chain thiols showed an 8-fold increase in response to nitrogen dioxide in comparison to bare Au-MWCNTs. Long-chain thiols having a hydrophilic head group were significantly less responsive to the species tested than their short-chain counterparts. Given the distance between neighboring Au nanoparticles, long-chain thiols collapse and overlap, generating a steric hindrance effect and favoring the formation of carboxylic acid dimers, which would explain the decreased sensitivity observed.

In a second step, the effect of ambient moisture on the response towards nitrogen dioxide was studied. It was observed that the presence of moisture adsorbed on the surface of the sensors increased the response of short-chain, hydrophilic thiol and carbon nanotube hybrids. This enhancement in the nitrogen dioxide response under humid conditions can be attributed to a water-mediated adsorption of nitrogen dioxide, already reported for semiconductor chemoresistors.

From an applications perspective, the range of nitrogen dioxide concentrations tested here (tens of ppm) is still too high for monitoring this species in ambient conditions (tens of ppb). However, there is room for improving these results, for example, by using single-walled carbon nanotubes instead of MWCNTs and by further optimizing the chain length, head functional groups and the amount of the carbon nanotube sidewall functionalization. Also, the time needed for recovering the baseline should be improved as well. A few well-known options to diminish the recovery time include heating or UV irradiating the gas-sensitive film during the recovery phase to ease desorption of molecules from the surface, increasing the flow rate during both detection and recovery phases or further optimizing the sensor parameters such as electrode design or CNT density.

According to the experimental results discussed here and in previously reported theoretical studies, the combination of hydrophilic and hydrophobic short-chain SAMs in Au-CNT sensor arrays should be helpful for detecting polar and nonpolar gases, even under fluctuating ambient moisture conditions.

## Experimental

### Functionalization of carbon nanotubes

Multiwall carbon nanotubes (MWCNTs) functionalized with carboxylic acid (COOH) were purchased from Nanocyl S.A. (Belgium). These nanotubes were produced by a catalytic chemical vapor deposition (c-CVD method) and purified to greater than 95%. The average length of these carbon nanotubes is 1.5 µm and the average diameter is 9.5 nm. The surface of the carbon nanotubes was modified with –COOH groups, with a value of mass greater than 8%. This surface modification was performed by Nanocyl S.A. via an oxygen-plasma treatment.

An attempt to decorate pristine CNTs with Au nanoparticles would lead to poor results [36]. Au is mobile on the surface of pristine CNTs, resulting in coalescence effects (uncontrolled size of particles and bad homogeneity in the decoration). Previous works have reported the benefits of employing oxygen-plasma-treated CNTs, which need to be subsequently decorated with Au nanoparticles. Oxygenated defects act as anchoring and nucleation sites. Therefore, high control in the decoration homogeneity and the size of Au nanoparticles (avoiding coalescence effects) have been reported in oxygen-plasma-treated CNTs [37]. Since oxygenated defects are homogeneously distributed in the CNTs used, we expect Au NPs to be homogeneously distributed on the whole surface, that is, not in direct contact to the substrate.

A suspension of these functionalized MWCNTs was prepared using *N*,*N*-dimethylformamide (DMF) purchased from Alfa Aesar (99.8% purity). Then, the suspension was placed in an ultrasonic bath during 30 min at room temperature. Thereafter, the suspension was deposited onto screen-printed alumina substrates by employing an airbrushing technique. Finally, the MWCNTs were decorated with gold nanoparticles by a sputtering method. An ATC Orion 8-HV sputtering machine (AJA International, Inc., USA) was used for this purpose. In this process, an RF inductively coupled plasma was used at a frequency of 13.56 MHz. The sputtering parameters were adjusted to 30 W under argon plasma during 10 s at a pressure of 0.1 Torr [50].

Au-decorated MWCNTs were further functionalized by employing SAMs of different thiols. Four thiols were used, namely, 1-propanethiol (C_3_H_8_S), 3-mercaptopropanoic acid (C_3_H_6_O_2_S), 1-hexadecanethiol (C_16_H_34_S) and 16-mercaptohexadecanoic acid (C_16_H_32_O_2_S), all purchased from Sigma-Aldrich. These thiols were chosen based on two main differences, the length of the carbon chain and the terminal functional group. Thiols with short (3 carbons) and long (16 carbons) length carbon chains were used. In addition, for each length of carbon chain, a hydrophilic functional group (–COOH) and other more hydrophobic (–CH_3_) groups were studied.

The affinity between sulfur and gold, which results in a strong bond [51], has been extensively reported. Therefore, sensors based on MWCNTs decorated with gold nanoparticles were immersed in a thiol solution. The different thiols employed were dissolved in ethanol to produce 0.1 mM solutions. Then, sensors consisting of mats of Au-decorated MWCNTs on alumina substrates were immersed in these solutions and kept at 4 °C during 24 h in order to ensure the correct formation of SAMs. Finally, the sensors were rinsed three times with ethanol and dried under a N_2_ flow to remove unbound thiols [28].

### Material characterization

The hydrophilicity of the gas-sensitive coatings was evaluated using an optical tensiometer from Biolin Scientific. Their chemical composition was characterized by Raman spectroscopy and X-ray photoelectron spectroscopy (XPS). Raman spectrometry was performed employing an instrument from Renishaw, Inc., (U.K.), which was coupled to a confocal Leica DM2500 microscope. The laser wavelength applied to the samples was 785 nm. The samples under inspection were held on a Peltier cell and kept at low temperature (4 °C) in order to ensure the stability of SAMs under laser irradiation. The chemical composition and, particularly, the presence of the Au–S bond were evaluated using a Versaprobe Phi 5000 instrument from Physical Electronics equipped with a monochromatic Al Kα X-ray source. The binding energy (BE) scales were referenced by setting the Au 4f_7/2_ to 84.0 eV. The XPS spectra were collected at a take-off angle of 45° with respect to the electron energy analyzer. The X-ray beam diameter was 200 µm. The energy resolution was 0.5 eV. For the compensation of built-up charge on the sample surface during the measurements, a dual-beam charge neutralization composed of an electron gun (1 eV) and an Ar ion gun (≤10 eV) was used.

The structural characterization of Au-decorated MWCNTs was performed using a JEM-1011 transmission electron microscope (TEM) from JEOL Ltd. (Japan). A copper grid was used onto which a suspension of Au-MWCNTs was dropped. The images were taken at 80 kV.

### Fabrication of the sensor substrate

A 10 × 10 mm^2^ alumina substrate was employed. Interdigitated electrodes and a heating resistor were screen-printed on either side of the substrate employing a platinum ink (see Supporting Information File 1, Figure S6). The electrode area was coated with a mat of CNTs via airbrushing through a shadow mask. Once the sensors were sputtered with gold and functionalized with thiols, two-wire contacts were made on the samples using a conductive epoxy (Ag component metallization, Heraeus) and platinum wires. The samples were bonded to a 20 × 30 mm^2^ printed circuit board (PCB), which was then plugged inside a test chamber (see Supporting Information File 1, Figure S7).

#### Gas sensing measurements

A 35 mL airtight test chamber in Teflon was connected to an automated gas mixture delivery system controlled by Bronkhorst mass-flow controllers and calibrated gas cylinders. The experimental conditions were set to deliver different concentrations of ethanol vapors and nitrogen dioxide gas at room temperature (20 °C) under a constant flow of 100 mL/min. Unless otherwise specified, the measurements were performed under dry conditions. For studying the effect of ambient moisture on the sensor response, the gas flow was humidified to a relative humidity of about 50%. The evolution of the sensor resistance was measured with an Agilent HP 34972A multimeter.

## Supporting Information

The chemical structure of the different thiols tested. Representation of self-assembled monolayers. Raman spectroscopy of carbon nanotubes. Deconvolution of the C 1s core level peak for Au-MWCNTs using XPS. Table with the relative abundance (%) analyzed by XPS technique. Contact angle measurements. Sensor fabrication process detailed. Alumina heater description and sensor wire-bonded to a PCB.

File 1 Figures related to thiol-Au-carbon nanotubes and experimental data obtained during the material characterization.
