# Supplementary material for: Hydrophilicity and carbon chain length effects on the gas sensing properties of chemoresistive, self-assembled monolayer carbon nanotube sensors
Source: Beilstein J Nanotechnol. 2019 Feb 27;10:565–77. doi: 10.3762/bjnano.10.58 (PMC6404396; doi:10.3762/bjnano.10.58)
Supplement: File 1 — Figures related to thiol-Au-carbon nanotubes and experimental data obtained during the material characterization. [file Beilstein_J_Nanotechnol-10-565-s001.pdf]

BEILSTEIN JOURNAL OF NANOTECHNOLOGY

Supporting Information

for

Hydrophilicity and carbon chain length effects on the gas sensing properties of chemoresistive, self-assembled monolayer carbon nanotube sensors

Juan Casanova-Cháfer, Carla Bittencourt and Eduard Llobet

Beilstein J. Nanotechnol. **2019,** *10,* 565–577. doi:10.3762/bjnano.10.58

Figures related to thiol-Au-carbon nanotubes and experimental data obtained during the material characterization

License and Terms: This is a supporting information file under the terms of the Creative Commons Attribution License (http://creativecommons.org/licenses/by/4.0). Please note that the reuse, redistribution and reproduction in particular requires that the authors and source are credited.
The license is subject to the *Beilstein Journal of Nanotechnology* terms and conditions: (https://www.beilstein-journals.org/bjnano)

Chemical structure of thiols

Four different thiols were tested differing in the length of the carbon chain and in the hydrophobic or hydrophilic nature of the functional group.

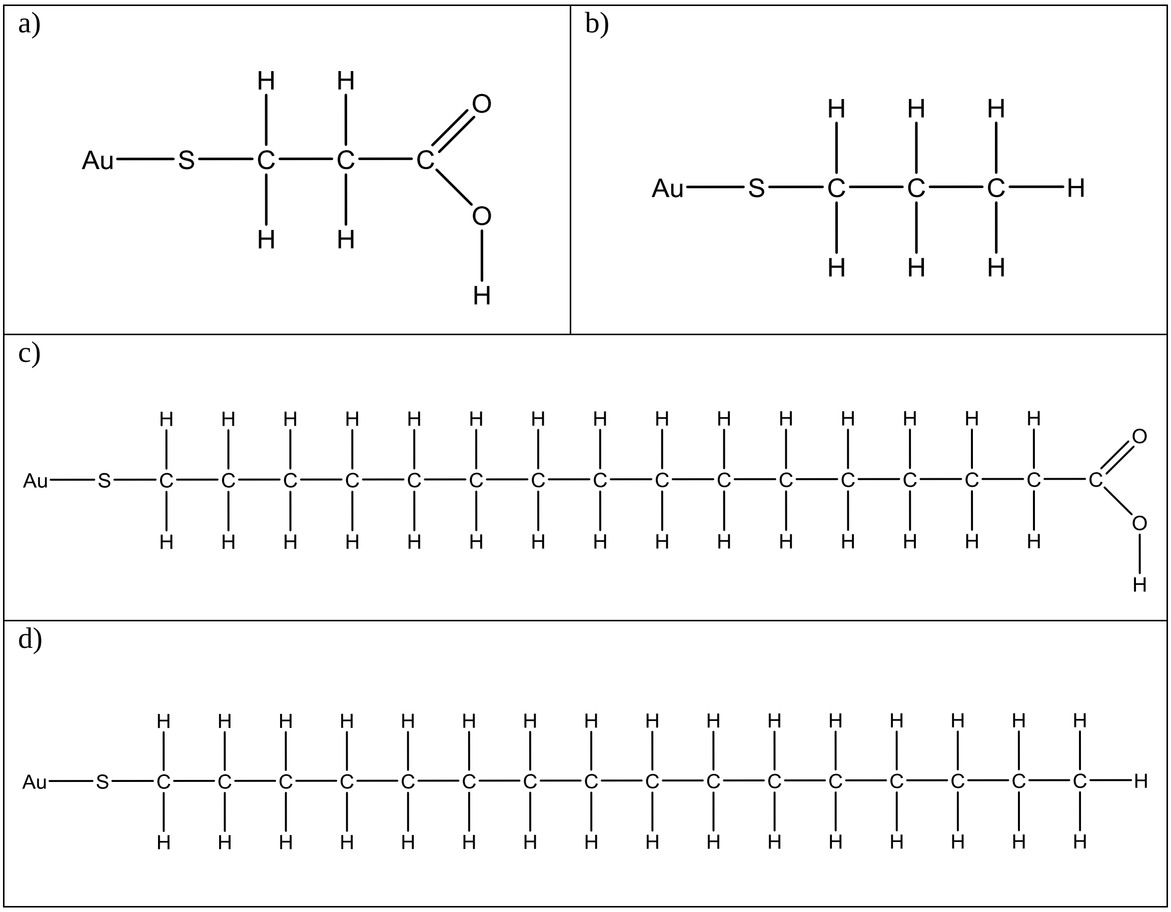

Figure S1: Chemical structure of $C_3H_6O_2S$ (a), C_3H_8S (b), $C_{16}H_{32}O_2S$ (c) and $C_{16}H_{34}S$ (d) bonded to gold nanoparticles.

Self-assembled monolayers

This figure illustrates the differences arising when carbon nanotubes are functionalized with short or long chain thiols.

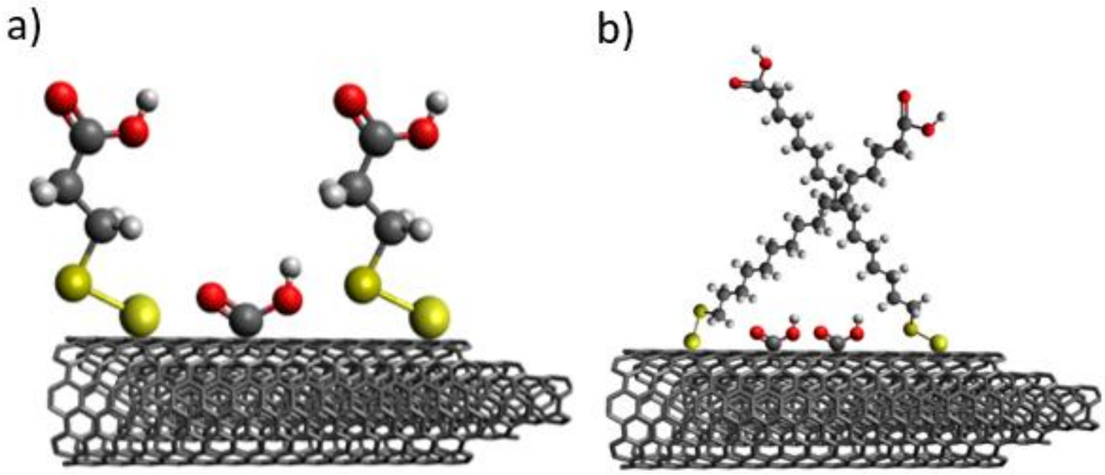

Figure S2: a) Short carbon length chain with exposed functional groups from both, SAMs and from MWCNT; b) Long carbon length chains overlap, reducing sensitivity and creating steric hindrance because gas molecules find more difficulties to access and interact with carboxylic groups from MWCNTs. Atom representation based on CPK color system: red = oxygen; black = carbon; white = hydrogen; dark yellow = sulfur; light yellow = gold.

Raman spectroscopy of carbon nanotubes

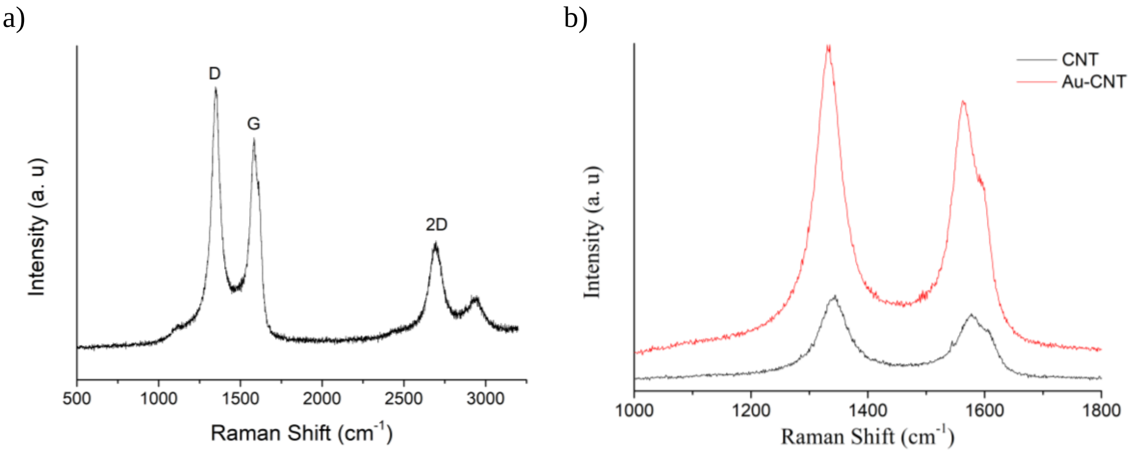

Figure S3: Raman spectra of MCNTs decorated with Au nanoparticles (a). Comparison of two Raman spectra in the D, G region for pristine and Au-decorated MWCNTs (b).

It is well-known that the Raman spectrum [1] of carbon nanotubes shows characteristic peaks at 1350 cm^{-1}, 1580 cm^{-1} and 2780 cm^{-1}, corresponding to the D, G and 2D bands, respectively (see Figure S3a). The presence of defects (carbonaceous impurities, broken sp^2 bonds, etc.) is

indicated by the D and 2D bands [2], meanwhile the G band represent the stretching of C-C bond in-plane oscillations of sp^2 in graphitic materials [3]. Besides, the Au-CNT hybrids show higher resolution of the bands, because the conductivity is improved by the presence of gold nanoparticles on the surface of MWCNTs [4].

In order to check the occurrence of LSPR [5] Raman spectra were acquired applying higher power to the laser on pristine and Au-MWCNT samples. Figure S3b shows the enhanced intensity of the Raman spectrum for Au-MWCNT samples, indicative of the LSPR effect caused by the presence of Au nanoparticles.

XPS analysis

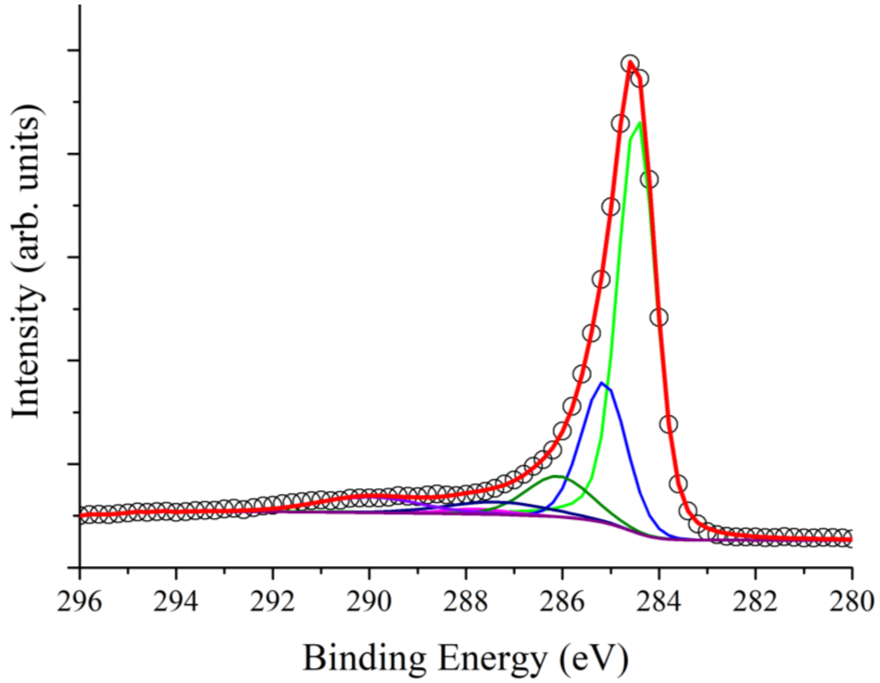

Figure S4: Deconvolution of the C 1s core level peak for Au-MWCNTs.

The chemical modification produced by the plasma treatment can be identified by a broad structure at high binding energy that can be attributed to photoelectrons emitted from carbon atoms belonging to hydroxyl (component centered at 286.2 eV) (8.7%), carbonyl or ether (component centered at 287.2 eV) (4.8%) and carboxyl or ester groups (component centered at 288.9 eV) [6] (5.8%). The peak at 285.1 eV can be associated to the presence of sp3 carbons (20.0 %). The % corresponds to the relative peak area of each component.

The evaporation of Au atoms onto plasma functionalized CNTs reduces the effective number of C 1s and O 1s photoelectrons contributing to the XPS spectra [6], as the Au nucleation centers occur mainly in the proximity of oxygenated defects created during the plasma treatment. The

contribution of photoelectrons emitted from oxygen atoms localized under the Au nanoparticles to the O 1s core level peak is expected to be smaller after the nanoparticles are covered with short thiols, because the kinetic energy of the O 1s photoelectron excited with photons of 1486 eV is ca. 960 eV (corresponding to an inelastic mean free path (IMFP) of ca. 20 A°). Therefore, due to the thiol + Au coverage, a significant decrease in the intensity of the O 1s peak is expected and thus, a reduction in the calculated relative amount of oxygen is obtained. A similar effect can explain the apparent reduction in the amount of Au at the CNT surface after the formation of SAMs.

Table S1: Relative abundance (%) of carbon, oxygen, sulfur and gold in either Au-MWCNT or SAM-Au-MWCNT samples.

**Sample**	**C [%]**	**O [%]**	**S [%]**	**Au [%]**
CNT + Au	88.3	6.0		5.7
CNT + Au + SAM	94.7	0.5	4.3	0.5

Hydrophilicity/hydrophobicity of the gas sensitive coatings

Figure S5 shows the results on contact angle measurement, which reveal the hydrophilicity of the surface samples. From left to right, the first image shows a contact angle near 70° for bare Au-MWCNTs, which are functionalized with COOH groups, giving this hydrophilic character to the surface. The image in the middle shows that a slightly lower contact angle (59°) is obtained for SAM-Au-MWCNT samples that present a hydrophilic functional group (COOH) as a head group. This decrease in contact angle is indicative that the hydrophilic properties are enhanced upon functionalization with hydrophilic thiols. Finally, the third image shows that a hydrophobic surface is achieved for SAM-Au-MWCNT samples that present a hydrophobic functional group (CH_3) as a head group. The contact angle for this SAM-Au-MWCNT sample is 104°.

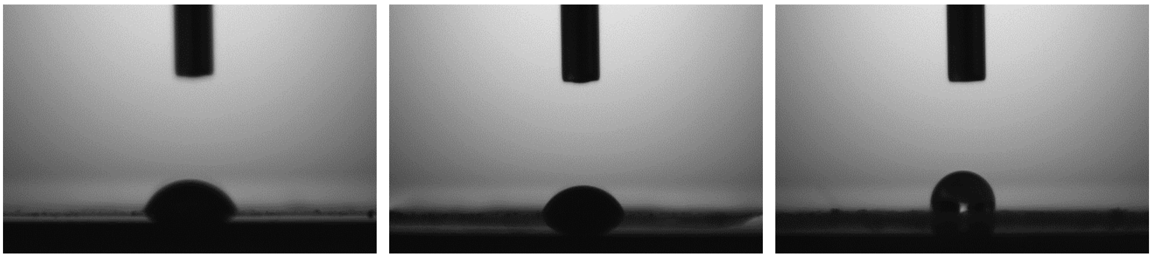

Figure S5: Contact angle measurements. From left to right: bare Au-MWCNTs functionalized with COOH groups, SAM-Au-MWCNTs sample with COOH functional group present in thiols and SAM-Au-MWCNTs sample with CH_3 head groups in thiols.

Sensor fabrication

Platinum ink was screen-printed onto alumina to produce the sensor substrates. On the front side interdigitated electrodes were printed. Electrode width was 500 µm and electrode gap was 400 µm. Electrode areas was 5 × 5 mm^2. A heating resistor (R = 7 ± 1 Ω @ R.T.) was printed on the backside of the substrate.

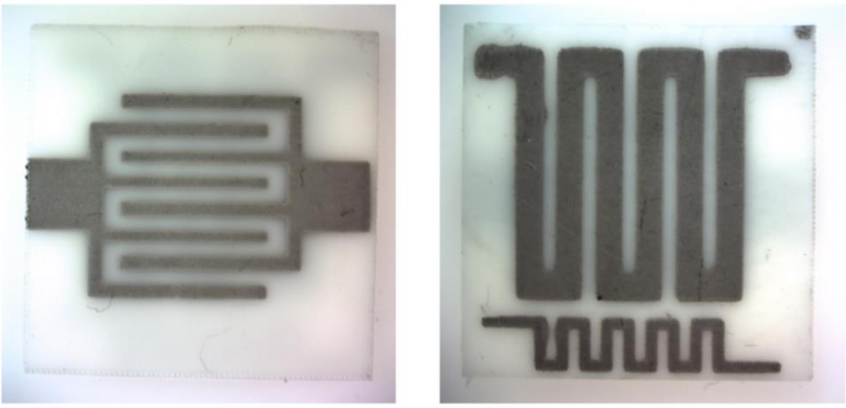

Figure S6: Double side screen-printed design onto alumina. Left side show the electrode used and right side display the heater applied. The size of the alumina substrate is 1 × 1 cm^2.

The picture below shows a sensor ready for testing. Once the carbon nanotube films are deposited by air brushing, covering the electrode area of the substrate through a shadow mask, and these are functionalized with thiols. Finally, substrates are wire bonded to a PCB that can be inserted into the test chamber for undergoing gas sensing tests.

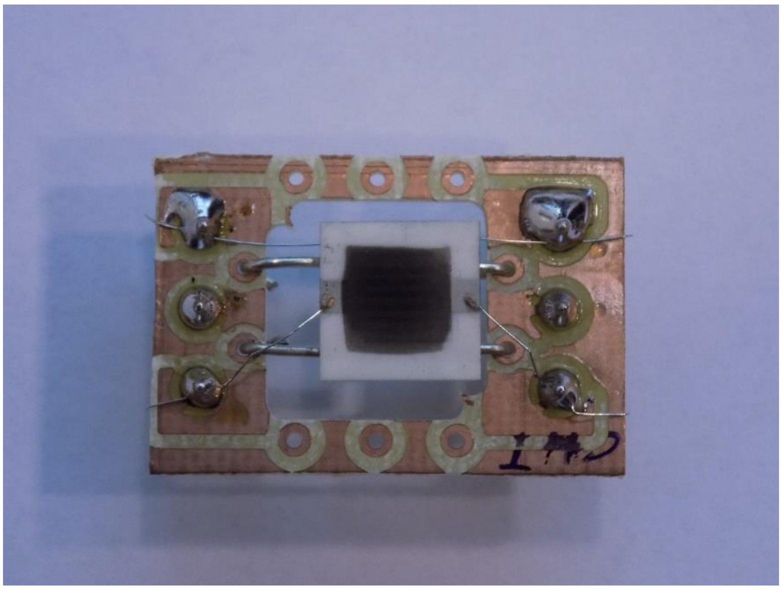

Figure S7: Picture of a sensor wire bonded to a PCB holder and ready for testing.

References

1. Wang, W.; Guo, S.; Ozkan, M.; Ozkan, C. *ECS Trans.* **2013,** *50,* 37–44. doi:10.1149/05043.0037ecst
2. Mudimela, P. R.; Scardamaglia, M.; González-León, O.; Reckinger, N.; Snyders, R.; Llobet, E.; Bittencourt, C.; Colomer, J. F. *Beilstein J. Nanotechnol.* **2014,** *5,* 910–918. doi:10.3762/bjnano.5.104
3. Eklund, P. C.; Holden, J. M.; Jishi, R. A. *Carbon* **1995,** *33*, 959–972. doi:10.1016/0008-6223(95)00035-C
4. Fernandes, C.; Pereira, C.; Guedes, A.; Rebelo, S. L. H.; Freire, C. *Appl. Catal., A* **2014,** *486,* 150–158. doi:10.1016/j.apcata.2014.08.031
5. Zhang, X.; Zhang, J.; Quan, J.; Wang, N.; Zhu, Y. *Analyst* **2016,** *141,* 5527–5534. doi:10.1039/c6an00850j
6. Suarez-Martinez, I.; Bittencourt, C.; Ke, X.; Felten, A.; Pireaux, J. J.; Ghijsen, J.; Drube, W.; Van Tendeloo, G.; Ewels, C. P. *Carbon* **2009,** *47,* 1549–1554. doi:10.1016/j.carbon.2009.02.002
